# Comparison of variant allele frequency and number of mutant molecules as units of measurement for circulating tumor DNA

**DOI:** 10.1002/1878-0261.12827

**Published:** 2020-10-31

**Authors:** Manouk K. Bos, Kazem Nasserinejad, Maurice P. H. M. Jansen, Lindsay Angus, Peggy N. Atmodimedjo, Evert de Jonge, Winand N. M. Dinjens, Ron H. N. van Schaik, Marzia Del Re, Hendrikus J. Dubbink, Stefan Sleijfer, John W. M. Martens

**Affiliations:** ^1^ Department of Medical Oncology Erasmus MC Cancer Institute, University Medical Center Rotterdam The Netherlands; ^2^ Department of Hematology HOVON Data Center Erasmus MC Cancer Institute, University Medical Center Rotterdam The Netherlands; ^3^ Department of Pathology Erasmus MC Cancer Institute, University Medical Center Rotterdam The Netherlands; ^4^ Department of Clinical Chemistry Erasmus University Medical Center Rotterdam The Netherlands; ^5^ Unit of Clinical Pharmacology and Pharmacogenetics Department of Clinical and Experimental Medicine University Hospital of Pisa Italy

**Keywords:** cancer, circulating tumor DNA, digital droplet PCR, liquid biopsy, next‐generation sequencing, unit of measurement

## Abstract

Quantification of tumor‐specific variants (TSVs) in cell‐free DNA is rapidly evolving as a prognostic and predictive tool in patients with cancer. Currently, both variant allele frequency (VAF) and number of mutant molecules per mL plasma are used as units of measurement to report those TSVs. However, it is unknown to what extent both units of measurement agree and what are the factors underlying an existing disagreement. To study the agreement between VAF and mutant molecules in current clinical studies, we analyzed 1116 TSVs from 338 patients identified with next‐generation sequencing (NGS) or digital droplet PCR (ddPCR). On different study cohorts, a Deming regression analysis was performed and its 95% prediction interval was used as surrogate for the limits of agreement between VAF and number of mutant molecules per mL and to identify outliers. VAF and number of mutant molecules per mL plasma yielded greater agreement when using ddPCR than NGS. In case of discordance between VAF and number of mutant molecules per mL, insufficient molecular coverage in NGS and high cell‐free DNA concentration were the main responsible factors. We propose several optimization steps needed to bring monitoring of TSVs in cell‐free DNA to its full potential.

## Graphical abstract

Here, we compare the number of mutant molecules and the variant allele frequency as units of measurement for circulating tumor DNA. We conclude that when using digital droplet PCR, both units of measurement yield greater agreement than when using next generation sequencing. Low frequent mutations and molecular coverage are important factors affecting agreement. These results will improve circulating tumor DNA analysis, contributing to better cancer patient management.

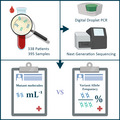

**Abbreviations**
µL: microliter
cfDNA: cell‐free DNA
CI: confidence interval
ctDNA: circulating tumor DNA
ddPCR: digital droplet PCR
EDTA: ethylenediaminetetraacetic acid
LOD: limit of detection
mL: milliliters
ng: nanograms
NGS: next‐generation sequencing
NPV: negative predictive value
PI: prediction interval
PPV: positive predictive value
TSV: tumor‐specific variant
UMI: unique molecular identifier
VAF: variant allele frequency

## 1 Introduction

The genomic characteristics of solid tumors increasingly determine how patients are being treated. Although metastatic tissue can be obtained for this analysis, it is a cumbersome procedure and thereby limits repetitive sampling. Molecular profiling of cell‐free DNA (cfDNA) in liquid biopsies from patients with cancer is evolving rapidly as a patient‐friendly tool to measure tumor load as well as to gain insight into tumor characteristics [1].

Although isolation of cfDNA from plasma is an easy procedure, DNA fragments from nonmalignant cells (e.g., lymphoid and myeloid cells) hamper the subsequent detection of tumor‐specific variants (TSVs) [2]. Generally, tumor‐derived cfDNA fragments (circulating tumor DNA or ctDNA) represent a minority of all cfDNA fragments present in plasma [3]. The lower limit of detection (LOD) of TSVs has been improved by digital droplet PCR (ddPCR), enabling the detection of tumor‐specific variants in a single DNA molecule [4]. In addition, the LOD of larger gene panels used for next‐generation sequencing (NGS) has been optimized by the development of unique molecular identifiers (UMIs). UMIs are added to each molecule before amplification, allowing correction of sequencing errors and identification of individual mutated templates [5, 6].

The quantity of a tumor‐specific variant is typically reported as the ratio between the number of mutated‐ and wild‐type DNA copies and is referred to as the variant allele frequency (VAF). During its clinical implementation, it has become clear that solely reporting the VAF does not suffice as it does not provide information on the concentration of a TSV. Use of a measurement that reports a concentration is common practice for other biomarkers, such as tumor antigens, as it is considered to reflect tumor load more adequately. Moreover, the cfDNA concentration is known to yield important prognostic value [7, 8, 9]. To this end, it might be preferable to use mutant molecules per mL plasma as a unit of measurement for monitoring of TSVs.

To what extent mutant copies per mL plasma relate to the VAF is currently unknown. To optimize characterization of cfDNA, including monitoring of TSVs in blood over time, information on agreement between VAF and mutant copies per mL plasma obtained by using real‐life data is pivotal. Here, we report the agreement between VAF and mutant copies per mL plasma as units of measurement for 1116 TSVs quantified by NGS or ddPCR using current‐day pipelines. Secondly, we identify pre‐analytical and analytical factors that hamper agreement between both units of measurement.

## 2 Methods

### 2.1 Sample collection

Samples were obtained from the following studies: IMPACT‐CRC study on colon cancer (ClinicalTrials.gov, number NCT02117466) [10], REGORA study on colon cancer (ClinicalTrials.gov, number NCT02800330), START‐TKI study on lung cancer (CCMO number, NL58664.078.16) [11], TAX‐ESR1 study on breast cancer (trialregister.nl, number NL7280), and the CareMore‐Trastuzumab study on breast cancer (trialregister.nl, number NL4977). The study was performed in accordance with the Declaration of Helsinki and approved by the medical ethics committee of the Erasmus MC. All patients gave written informed consent prior to study procedures.

Samples from patients with lung cancer were collected upon progression on current therapy for detection of primary activating and p.T790M *EGFR* mutations. Samples from patients with colon cancer were collected before start and during anti‐*EGFR* therapy or before start and during regorafenib therapy. Samples from patients with breast cancer were collected before start of first‐line taxane‐based chemotherapy. For some patients with colon cancer, serially collected samples were available. Blood was collected in either EDTA or CellSave tubes. For blood collected in EDTA tubes, plasma was separated within 4 h as recommended by the latest guidelines [12, 13].

### 2.2 Description of included cohorts: cfDNA isolation and quantification

The pre‐analytical work‐up of samples within a study cohort were consistent and are described below.

#### 2.2.1 Cohort 1

Patients with metastatic colon cancer. CfDNA was isolated using the QiaSymphony Circulating DNA kit (Qiagen, Venlo, the Netherlands) from 950 to 4000 µL plasma and eluted in 70 µL elution buffer. Depending on the amount of cfDNA, 20 ng was used for NGS. Samples were concentrated using a speedvac concentrator if necessary. For a subset of patients, longitudinal ddPCR data were available. Generally, 7 µL eluate or less was used for ddPCR, depending on the eluate concentration. The maximum input was 50 ng, and the median input was 12 ng.

#### 2.2.2 Cohort 2

Patients with lung cancer. CfDNA was isolated from 3 mL plasma with the QIAGEN QIAamp Circulating Nucleic Acid kit (Qiagen) and eluted in 50 µL elution buffer. For NGS, the samples were then concentrated to 25 µL using a Speedvac concentrator before cfDNA was quantified by Qubit fluorometer (Invitrogen, Carlsbad, CA, USA). Subsequently, 13 µL of concentrated sample with a maximum of 50 ng was used for NGS. For a subset of patients, ddPCR data were available. For ddPCR, 4 µL of the unconcentrated eluate was used regardless of cfDNA concentration.

#### 2.2.3 Cohort 3

Patients with metastatic breast cancer. CfDNA was isolated from 600 to 4000 µL plasma using the QIAGEN QIAamp Circulating Nucleic Acid kit (Qiagen) and eluted in 50 µL elution buffer. Depending on the amount of cfDNA 10 ng was used for NGS. Samples were concentrated using a speedvac concentrator if necessary. No ddPCR data were available for this cohort.

For all samples across cohorts, cfDNA concentrations were measured using the Quant‐iT dsDNA high‐sensitivity assay (Invitrogen, Life Technologies, Carlsbad, CA, USA) according to the manufacturer's instructions, and the Qubit fluorometer (Invitrogen) was used as read out.

### 2.3 Next‐generation sequencing

All samples were sequenced with the Ion Torrent™ Oncomine™ cfDNA Assay for breast, colon or lung cancer for the respective cancer type, on the Ion Torrent S5XL Next Generation Sequencing (NGS) prime system, all according to protocols and consumables provided by the manufacturer [10, 11] (Life Technologies, Thermo Fisher Scientific, Carlsbad, CA, USA). NGS panels were equipped with UMIs to enable detection of unique mutated copies. Molecular coverage was defined as the count of unique molecules and known hotspot variants were analyzed if they were detected in at least three independent molecules. Additionally, variants detected in cohort 1 and cohort 3 were called as true variants either if the molecular coverage was sufficient given the input of cfDNA (a minimum of 500 unique molecules for each 10 ng of DNA sequenced), or if the variant was detected with sufficient coverage in an earlier sample from the same patient.

### 2.4 ddPCR analysis

Analysis of mutations was performed using uniplex ddPCR mutation assays from Bio‐Rad Laboratories or Thermo Fisher as previously described [11, 14]. Variants were designated true variants if they were detected in at least three independent molecules. The number of droplets positive for mutant or wild‐type molecules was fitted into a Poisson distribution to determine the absolute number of copies per µL eluate, thereby correcting for droplets containing more than one molecule. The number of mutant molecules was then derived from the concentration of mutant copies per µL eluate. The VAF was reported by the Bio‐Rad software, after correction for the Poisson distribution. Molecular coverage was calculated as the sum of wild‐type and mutant copies.

### 2.5 Data collection and definitions

Data on sequencing input, isolation protocol, Qubit measurements, amount of plasma used for isolation, molecular coverage, and number of mutant copies were collected. VAF was calculated as (number of mutant copies/(number of wild‐type copies + number of mutant copies)) × 100% and the number of mutant copies per mL plasma was calculated follows, for NGS: (number of mutant copies/DNA input for sequencing (ng)) × (cfDNA concentration (ng·mL^−1^ plasma)), and for ddPCR: (number of mutant copies/input for analysis (μL)) × (total eluate (μL)/amount of plasma used for isolation (mL)). As a result, the multiplication factor by which the number of mutant copies are multiplied to calculate the number of mutant molecules per mL plasma was defined as the ratio between the cfDNA concentration and the cfDNA assay input: cfDNA concentration (ng·mL^−1^ plasma)/cfDNA sequencing input (ng) (NGS) or Total eluate (μL)/(Assay input (μL) × Total amount of plasma used for isolation (mL)) (ddPCR).

### 2.6 Statistics

To assess the agreement between VAF and mutant molecules per mL plasma, a Deming regression analysis was performed and its 95% prediction interval (PI) was calculated. The average width of the 95% prediction interval was used as a surrogate for the 95% limits of agreement. This method is adopted from Bland and Altman [15], which is used to study agreement between measurements with different units. The average width of the 95% PI is an important measure that indicates whether it is clinically acceptable to replace one method by another. TSVs outside the 95% PI were considered outliers, in which TSVs that lie above the 95% PI upper limit were considered as upper limit outliers, whereas TSVs that lie below the 95% lower limit were considered lower limit outliers, for *x* = VAF and *y* = mutant copies per mL plasma.

To assess the association between cfDNA concentration and molecular coverage for both VAF and mutant copies per mL, we calculated Pearson correlation using linear regression.

For NGS, we analyzed the within‐sample read coverage and molecular coverage of the different hotspots among the breast panel in cohort 3. Those samples were sequenced in different sequencing runs. The coefficient of variation for both the read coverage and the molecular coverage of different hotspots within a unique sample was reported. The coverage was normalized for cfDNA input using the total number of reads within a sample. Additionally, we calculated the positive and negative predictive value (PPV and NPV, respectively) of the factor molecular coverage < 500 molecules for lower limit outliers.

To analyze the correlation between pre‐analytical and analytical factors, Pearson correlation was calculated. All variables were log transformed before analysis.

Descriptive statistics were performed using ibm spss statistics 25 (IBM Corp, Armonk, NY, USA). All regression computations and graphics were performed in r program language [16]. Because the different cohorts included in this analyses were heterogeneous with regard to the pre‐analytical work‐up, we performed statistical analyses on each cohort separately. Thereby, our estimates of agreement approximated the real‐life setting.

## 3 Results

### 3.1 Description of samples and pre‐analytical variables

In total, 845 TSVs identified with NGS and 271 TSVs identified with ddPCR from 338 unique patients with solid tumors were included in this analysis. The cohort size was 53, 268, and 17 patients for, respectively, cohort 1 (colon), cohort 2 (lung), and cohort 3 (breast). Analyses using ddPCR were performed on a subset of samples from cohort 1 and 2 of whom NGS data were available. For cohort 1, additional longitudinal ddPCR analyses were performed. The median cfDNA concentration was 43 ng·mL^−1^ (range: 5–568 ng·mL^−1^), 13 ng·mL^−1^ (range: 4–452 ng·mL^−1^), and 21 ng·mL^−1^ (range: 8–58 ng·mL^−1^) for samples in cohort 1, 2, and 3, respectively (Table 1).

**Table 1:** Characteristics of samples included in this study. QS, QIAsymphony; QA, QIAamp.

Variable	NGS			ddPCR	
	Cohort 1	Cohort 2	Cohort 3	Cohort 1	Cohort 2
Tumor type	Colon	Lung	Breast	Colon	Lung
Number of patients	53	268	17	17	130
Number of samples	93	271	17	74	130
Baseline	44	271	17	19	130
Follow‐up	49	0	0	55	0
Number of TSVs	313	499	33	74	197
Blood collection tube type	EDTA	CellSave	CellSave	EDTA	CellSave
Isolation platform	QS	QA	QA	QS	QA
Sequencing assay	Oncomine Colon	Oncomine Lung	Oncomine Breast	KRAS G12G/V, PIK3CA E545K, TP53 various assays	EGFR L858R/T790M/Ex19Del
Plasma input for isolation (μL)					
Median	3600	3000	3400	2300	3000
Range	1700–4000	3000–3000	600–4000	950–3500	3000–3000
cfDNA concentration (ng·mL^−1^)					
Median	43	13	21	62	14
Range	5–568	3–452	8–58	11–670	3–277
Sequencing input (ng)					
Median	20	20	10	12	3
Range	11–24	4–50	10–10	1–54	1–64
Molecular coverage					
Median	2908	1917	1397	1887	477
Range	137–9307	169–17 317	269–3204	67–8410	43–6627
Multiplication factor					
Median	2.15	0.64	2.06	5	4.17
Range	0.33–28.41	0.64–4.17	0.83–5.84	2.64–36.06	4.17–6.25

### 3.2 Agreement analysis

**Fig. 1:**
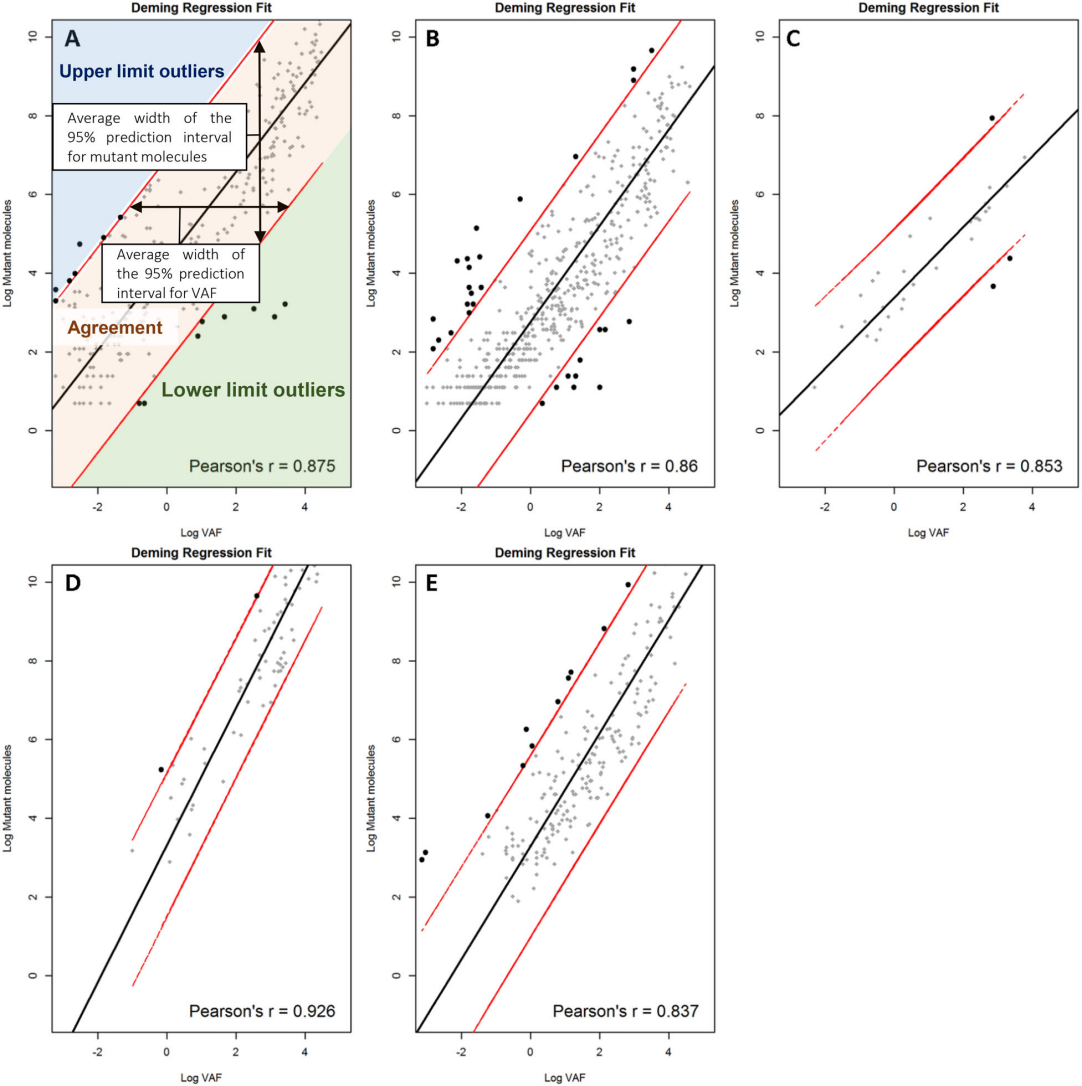
Deming regression analysis on tumor‐specific variants (TSVs) identified by Next Generation Sequencing (NGS; A–C) and digital droplet PCR (ddPCR; D, E). Red lines indicate the 95% prediction interval. (A) Cohort 1, NGS. Average width of the prediction interval for VAF = 99%, mutant copies = 183 copies. (B) Cohort 2, NGS. Average width of the prediction interval for VAF = 44%, mutant copies = 104 copies. (C) Cohort 3, NGS. Average width of the prediction interval for VAF = 49%, mutant copies = 33 copies. (D) Cohort 1, ddPCR. Average width of the prediction interval for VAF = 8%, mutant copies = 34 copies. (E) Cohort 2, ddPCR. Average width of the prediction interval for VAF = 24%, mutant copies = 100 copies.

For NGS, 95%, 94%, and 91% of all TSVs yielded agreement between VAF and mutant molecules per mL in cohort 1, 2, and 3, respectively. The Pearson's *r* was 0.875 [95% confidence interval (95% CI): 0.821–0.929] for cohort 1, 0.860 (95% CI: 0.815–1.000) for cohort 2 and 0.853 (95% CI: 0.659–1.000) for cohort 3 (Fig. 1A–C). For ddPCR, 97% and 94% of all TSVs yielded agreement within cohort 1 and 2. The Pearson's *r* was 0.926 (95% CI: 0.837–1.000) and 0.837 (95% CI: 0.759–0.914) for these samples. No lower limit outliers were detected within TSVs that were identified with ddPCR (Fig. 1D,E). The average width of the 95% PI was smaller for ddPCR than for NGS (cohort 1: VAF 8% for ddPCR vs 99% for NGS and mutant molecules 33 for ddPCR vs 183 molecules for NGS; cohort 2: 24% for ddPCR vs 44% for NGS and 100 for ddPCR vs 104 molecules for NGS, respectively, Fig. 1A,B/D,E). Hence, TSVs identified with ddPCR showed greater agreement between VAF and mutant molecules.

Outliers were identified in samples with both single and multiple TSVs. In some cases, all TSVs within a sample were outliers, whereas in other cases, samples contained TSVs that were either outlier or showed agreement (Table 2A,B, Table S2).

**Table 2:** Distributions of (pre‐)analytical factors among TSVs with agreement and outliers identified by (A) NGS and (B) ddPCR. Results are presented as median (range) unless indicated otherwise. When ≤ 2 outliers are present, only a range is given. Multiplication factor: cfDNA concentration (ng·mL^−1^)/Sequencing input (ng), TSV, tumor‐specific variant; NP, not present, no lower limit outliers were present in ddPCR.

A			
Variable	Agreement	Lower limit outlier	Upper limit outlier
Number of TSVs			
Cohort 1	*N* = 296	*N* = 8	*N* = 8
Cohort 2	*N* = 467	*N* = 10	*N* = 21
Cohort 3	*N* = 29	*N* = 2	*N* = 1
Number of samples			
Cohort 1	*N* = 91	*N* = 5	*N* = 2
Cohort 2	*N* = 261	*N* = 8	*N* = 11
Cohort 3	*N* = 15	*N* = 1	*N* = 1
Molecular coverage			
Cohort 1	3145 (436–9444)	424 (62–889)	8512 (3029–9641)
Cohort 2	2090 (169–22 905)	203 (68–257)	9202 (3224–22 919)
Cohort 3	1336 (584–3268)	269–338	2842
cfDNA concentration (ng·mL^−1^)			
Cohort 1	54 (5–568)	19 (14–31)	218 (218–568)
Cohort 2	14 (3–452)	4 (3–34)	192 (17–353)
Cohort 3	20 (9–45)	8–8	58
Multiplication factor			
Cohort 1	2.73 (0.33–28.41)	0.93 (.060–1.47)	9.0 (9.0–28.41)
Cohort 2	0.64 (0.64–4.17)	0.64 (0.64–0.69)	4.17 (0.64–4.17)
Cohort 3	2.02 (0.86–4.50)	0.83–0.83	5.85
Sequencing input (ng)			
Cohort 1	20 (11–24)	21 (20–23)	24 (20–24)
Cohort 2	20 (4–64)	6 (4–50)	47 (6–50)
Cohort 3	10 (10–10)	10–10	10

B			
Variable	Agreement	Lower limit outlier	Upper limit outlier
Number of TSVs			
Cohort 1	*N* = 72	*N* = 0	*N* = 2
Cohort 2	*N* = 186	*N* = 0	*N* = 11
Number of samples			
Cohort 1	*N* = 72	*N* = 0	*N* = 2
Cohort 2	*N* = 124	*N* = 0	*N* = 6
Molecular coverage			
Cohort 1	1835 (67–8477)	NP	2260–3525
Cohort 2	465 (43–6627)	NP	1044 (129–8648)
cfDNA concentration (ng·mL^−1^)			
Cohort 1	59 (11–670)	NP	92–210
Cohort 2	13 (3–268)	NP	118 (9–192)
Multiplication factor			
Cohort 1	5.21 (2.64–30.06)	NP	4.94 – 11.31
Cohort 2	4.17 (4.17–4.17)	NP	4.17 (4.17–6.25)
Sequencing input (ng)			
Cohort 1	12 (1–54)	NP	14–32
Cohort 2	3 (1–64)	NP	28 (2–46)

### 3.3 Distribution of pre‐analytical and analytical variables among outliers

For NGS, the cfDNA concentration and molecular coverage were higher in upper limit outliers compared to TSVs showing agreement (cfDNA concentration median, cohort 1: 218 vs 54 ng·mL^−1^; cohort 2: 192 vs 14 ng·mL^−1^; cohort 3: 58 vs 20 ng·mL^−1^) (molecular coverage median, cohort 1: 8512 vs 3145X; cohort 2: 9202 vs 2090X; cohort 3: 2842 vs 1338X) (Table 2A). Only in samples from cohort 2, the nanogram cfDNA input was higher among upper limit outliers (47 vs 20 ng). However, cfDNA input strongly correlated with cfDNA concentration and molecular coverage in this cohort since a fixed eluate volume was used for sequencing (Pearson's *r*: 0.600 and 0.606) (Table S1A). Additionally, both molecular coverage and cfDNA concentration were lower among lower limit outliers than in TSVs with agreement for all cohorts (molecular coverage median; cohort 1: 424 vs 3145X; cohort 2: 203 vs 2090X; cohort 3: 269–338 vs 1338X) (cfDNA concentration median; cohort 1: 19 vs 54 ng·mL^−1^; cohort 2: 4 vs 14 ng·mL^−1^; cohort 3: 8 vs 20 ng·mL^−1^). Although the sequencing input from samples in cohort 1 did not substantially differ between upper or lower limit outliers and samples that showed agreement, both cfDNA concentrations and molecular coverage were lower in lower limit outliers and higher in upper limit outliers compared to TSVs with agreement.

For ddPCR, no lower limit outliers were detected (Table 2B). Nanogram input, molecular coverage, and cfDNA concentration were higher in upper limit outliers in all cohorts. However, ddPCR all these variables were however strongly correlated with each other (Table S1B).

### 3.4 Molecular coverage in NGS and its impact on agreement

**Fig. 2:**
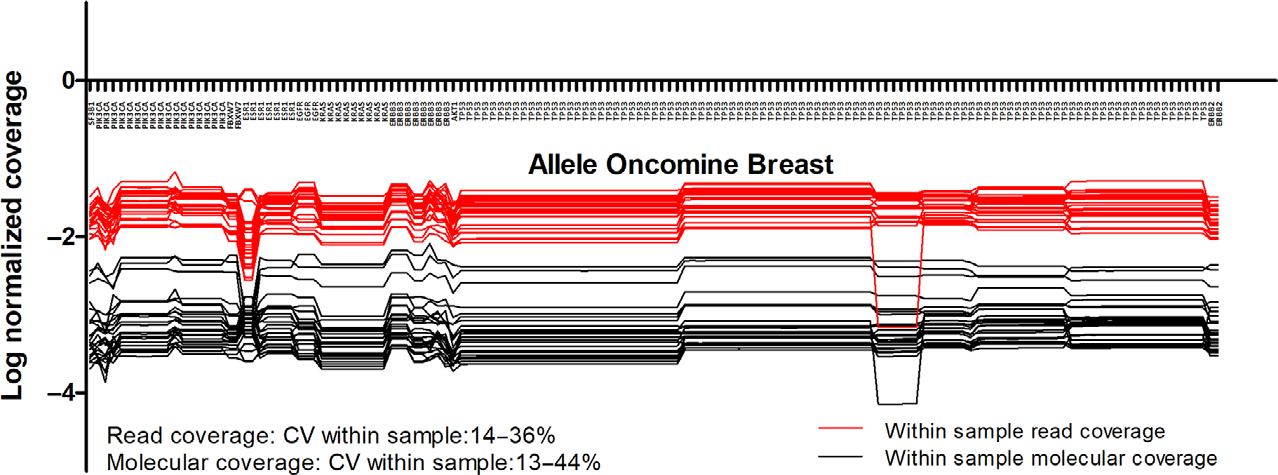
Within‐sample read coverage and molecular coverage per amplicon in Oncomine Breast panel. The molecular coverage was normalized for the total number of reads within a sample.

In lower limit outliers, the molecular coverage was substantially lower compared to samples showing agreement. A molecular coverage < 500 molecules, set arbitrary, resulted in a PPV of 0.55–1 and a NPV of 0.99–1 for lower limit outliers among all NGS detected TSVs. The median coefficient of variation (CV) of the read coverage and molecular coverage among different amplicons within a sample were 21% and 18% for the breast panel, respectively. The maximum observed CV within a sample was 36% for the read coverage and 44% for the molecular coverage. The coverage of gene positions located on the same amplicon did not vary. However, the coverage of amplicons within genes and across genes was highly divergent (Fig. 2).

## 4 Discussion

The use of ctDNA as a biomarker for real‐time monitoring of disease burden in a minimally invasive way is a promising tool to evaluate TSVs in plasma of patients with solid tumors. Although the importance of ctDNA load in plasma has been recognized before, its quantification is still in its infancy as demonstrated by the different units of measurements that are currently used to report those TSVs. In our analyses, we assessed agreement between VAF and mutant copies per mL and determined factors that affect this agreement. Here, we propose several optimization steps that result from these analyses.

Firstly, our analyses demonstrate that a low molecular coverage resulted in a severe underestimation of the absolute number of mutant molecules or an overestimation of the VAF. Assuming the sequencing efficiency for both mutant and wild‐type copies is equal, insufficient molecular coverage would affect the VAF to a lesser extent. A molecular coverage < 500 molecules in NGS had a high NPV for lower limit outliers indicating that a molecular coverage of > 500 molecules would assure a correct interpretation of the number of mutant molecules per mL plasma. To this end, our results suggest that sequencing quality controls should incorporate a minimum threshold for molecular coverage when TSVs are reported as mutant copies per mL.

In addition, we demonstrate that the molecular coverage in NGS is highly variable among amplicons present in a panel. For some individual samples, the within‐run CV for different amplicons was as high as 44%. For different variants within a sample with similar VAFs this would result in nearly a doubling of the number of mutant molecules per mL, solely based on the molecular coverage of certain positions. As the read coverage was impacted by a very similar variation, this observation is mainly attributable to variability in sequencing efficiency and less likely from UMI adapter ligation and subsequent read loss and decreased read quality [17, 18]. Lower limit outliers resulting from insufficient molecular coverage were identified in both samples with single and multiple TSVs. In some samples, only one amplicon was affected by low sequencing efficiency, whereas in other samples the molecular coverage was below 500 molecules for all amplicons. Amplicons in the genes *TP53* and *EGFR* were most frequently affected by sequencing efficiency (Table S2). In this study, we have used sequencing data generated by the use of Oncomine amplicon‐based panels designed for the IonTorrent sequencer as these panels are commonly used in the diagnostic facility of our Pathology department. For both amplicon and capture‐based panels, the molecular coverage is subjected to variation. Capture‐based panels can isolate neighboring regions that are not of interest, which will result in lower overall coverage in the regions of interest. Contrarily, amplicon‐based panels are subjected to issues related to primer design. Single nucleotide polymorphisms and short indels in the primer template region might cause allelic dropout resulting in decreased molecular coverage, whereas amplification of genes with high guanine‐cytosine content will less likely be effective [19]. To overcome this problem, NGS panels should be equipped with adequate quality controls to enable correction for molecular coverage per amplicon after sequencing [20].

Another factor affecting agreement between VAF and mutant molecules per mL plasma was the cfDNA concentration. A high cfDNA concentration was associated with upper limit outliers. However, not all TSVs in samples with a high cfDNA concentration were upper limit outliers, they were mainly TSVs with a frequency of < 0.01% (Table S2). Low frequent TSVs (i.e. TSVs detected in a low absolute number of mutant copies) are more frequently prone to stochastic errors when calculating the number of mutant molecules that originate from 1 mL plasma. Alternatively, stochastic errors that occur during amplification favored by the abundance of wild‐type molecules might result in an underestimation of the VAF.

In our analysis, quantification of TSVs by ddPCR yielded less outliers and smaller limits of agreement for mutant copies per mL. In addition, the molecular coverage was always sufficient to estimate mutant molecules per mL plasma resulting in the absence of lower limit outliers. Our analysis does not substantiate whether a fixed input based on eluate volume is superior to input based on cfDNA amount. Although not directly substantiated by our data, it is well known that the molecular coverage is an important determinant of the limit of detection of a TSV [19, 20]. To avoid false‐negative results, the molecular coverage should be sufficient to detect a TSV of a given frequency. To this end, the molecular coverage is an important parameter and should be reported for proper interpretation of sequencing results.

Although our analyses were limited by the inability to analyze the accuracy of all pre‐analytical and analytical steps and their impact on agreement separately, it does provide insights into the agreement of both units of measurement in the current real‐life setting (Table 3). Although our primary aim was to investigate agreement between VAF and mutant molecules for different sequencing platforms, we lacked power to investigate differences between pre‐analytical and analytical factors among outliers. We therefore only used descriptive statistics to describe differences among pre‐analytical and analytical variables between outliers and TSVs that showed agreement. For molecular coverage however, we were able to identify a threshold that showed a high NPV. Additionally, we did not include DNA from leukocytes to exclude germline variants or variants resulting from clonal hematopoiesis [2]. Although clonal hematopoiesis is known to occur in specific genes and we reported predominantly activating, cancer‐specific hotspot mutations, a nonmalignant origin of reported TSVs cannot be excluded. All samples from cohorts that were included in this study were collected for research purposes and pre‐analytical and analytical methods were homogeneous within study cohorts. The methods used in all cohorts are in agreement with current guidelines and recent literature. To this end, results from our analyses reflect the accuracy of the real‐life analyses pipeline as a whole.

**Table 3:** Overview of factors affecting VAF and/or mutant molecules per mL plasma.

Absolute count	Ratio	
More accurate representation of tumor burden	Less accurate representation of tumor burden	
Dependent of cfDNA isolation efficiency	Independent of cfDNA isolation efficiency	Assuming between‐run isolation efficiency affects both mutant and wild‐type molecules equally within isolation platforms. Between‐run isolation efficiency, and therefore cfDNA concentrations, are variable especially for manual isolation platforms
Directly affected by molecular coverage	Affected by molecular coverage if frequency of TSV is low; that is, mutant copies are disproportionally affected by stochastic errors	In NGS, a minimal molecular coverage of at least 500 molecules is advised to correctly calculate the number of mutant molecules
Dependent of cfDNA input if frequency of TSV is low; that is, mutant copies are disproportionally affected by stochastic errors	Dependent of cfDNA input if frequency of TSV is low; that is, mutant copies are disproportionally affected by stochastic errors	The LOD of TSVs decreases with increasing contamination by wild‐type molecules
Absolute count	Ratio	

## 5 Conclusions

From our study, we conclude that VAF and number of mutant molecules per mL plasma yielded greater agreement when using ddPCR than when using NGS. Given the higher costs of NGS, ddPCR might be preferred in case a specific variant can be tracked. For clinical purposes, the unit of measurement should reflect the concentration of cfDNA, alike other biomarkers. However, we demonstrate that quantification of absolute numbers of molecules per mL plasma is currently more heavily affected by pre‐analytical and analytical variables than the VAF. To this end, standardization of methods is necessary. Based on these analyses, we propose several optimization steps to be taken with respect to the isolation and quantification of cfDNA but also for NGS panels, to bring longitudinal monitoring of TSVs in cfDNA to its full potential.

## Conflict of interest

Manouk K Bos – Research funding: Dutch Cancer Society (no. NKB‐EMCR‐2016‐108154). Maurice P. H. Jansen – Research funding: Dutch Cancer Society (no. NKB‐NKI‐2014‐7080). Lindsay Angus – Consulting honorarium: Merck; Speaking honorarium: Pfizer. Winand N.M. Dinjens – Consulting honorarium: Bristol‐Myers Squibb, Roche, Bayer, Astra Zeneca, Novartis. Marzia del Re – Speaker honoraria: Astellas, Astra Zeneca, Celgene, Novartis, Pfizer, Bio‐Rad, Janssen, Sanofi‐Aventis; Consulting honoraria: Ipsen, Janssen‐Cilag, Sanofi‐Aventis; Speaker's bureau: Celgene, Janssen, Sanofi; Travel support: Astra‐Zeneca, Celgene, Janssen, Bio‐Rad. Hendrikus Jan Dubbink– Supported by a grant from AstraZeneca; Consulting or Advisory Role: AstraZeneca. John W.M. Martens – Consulting honorarium: Novartis; Research funding: Cancer Genomics Netherlands (CGC.nl), funded by the Netherlands Organization for Scientific Research (NWO). Kazem Nasserinejad, Peggy Atmodimedjo, Evert de Jonge, Ron H.N. van Schaik, Stefan Sleijfer – No conflict of interest.

## Author contributions

MKB wrote the manuscript that was corrected and approved by KN, MPHMJ, LA, PNA, EJ, WNMD, RHNS, MDR, HJD, SS, and JWMM. KN performed all statistical analyses.

## Supporting information


**Table S1.** (A) Correlation among (pre)analytical factors in NGS. (B) Correlation among (pre)analytical factors in ddPCR
**Table S2.** Pre‐analytical variables in samples containing outliers

## References

[1] Heitzer E (2019) Circulating tumor DNA for modern cancer management. Clin Chem 66, 143–145. 10.1373/clinchem.2019.304774 31672857

[2] Razavi P , Li BT , Hou C , Shen R , Venn O , Lim RS , Hubbell E , De Bruijn I , Liu Q , Satya RV *et al* (2017) Cell‐free DNA (cfDNA) mutations from clonal hematopoiesis: Implications for interpretation of liquid biopsy tests. J Clin Oncol 35, 11526.

[3] Wan JCM , Massie C , Garcia‐Corbacho J , Mouliere F , Brenton JD , Caldas C , Pacey S , Baird R & Rosenfeld N (2017) Liquid biopsies come of age: towards implementation of circulating tumour DNA. Nat Rev Cancer 17, 223–238. 28233803 10.1038/nrc.2017.7

[4] Vogelstein B & Kinzler KW (1999) Digital PCR. Proc Natl Acad Sci USA 96, 9236–9241. 10430926 10.1073/pnas.96.16.9236 PMC17763

[5] Newman AM , Bratman SV , To J , Wynne JF , Eclov NC , Modlin LA , Liu CL , Neal JW , Wakelee HA , Merritt RE *et al* (2014) An ultrasensitive method for quantitating circulating tumor DNA with broad patient coverage. Nat Med 20, 548–554. 24705333 10.1038/nm.3519 PMC4016134

[6] Smith T , Heger A & Sudbery I (2017) UMI‐tools: modeling sequencing errors in unique molecular Identifiers to improve quantification accuracy. Genome Res 27, 491–499. 28100584 10.1101/gr.209601.116 PMC5340976

[7] Dawson SJ , Tsui DW , Murtaza M , Biggs H , Rueda OM , Chin SF , Dunning MJ , Gale D , Forshew T , Mahler‐Araujo B *et al* (2013) Analysis of circulating tumor DNA to monitor metastatic breast cancer. N Engl J Med 368, 1199–1209. 23484797 10.1056/NEJMoa1213261

[8] Lecomte T , Berger A , Zinzindohoue F , Micard S , Landi B , Blons H , Beaune P , Cugnenc PH & Laurent‐Puig P (2002) Detection of free‐circulating tumor‐associated DNA in plasma of colorectal cancer patients and its association with prognosis. Int J Cancer 100, 542–548. 12124803 10.1002/ijc.10526

[9] Viller Tuxen I , Barlebo Ahlborn L , Mau‐Soerensen M , Staal Rohrberg K , Cilius Nielsen F , Oestrup O , Westmose Yde C , Richter Vogelius I & Lassen U (2019) Plasma total cell‐free DNA is a prognostic biomarker of overall survival in metastatic solid tumour patients. Br J Cancer 121, 125–130. 31186525 10.1038/s41416-019-0491-9 PMC6738043

[10] van Helden EJ , Angus L , der Houven M‐V , van Oordt CW , Heideman DAM , Boon E , van Es SC , Radema SA , van Herpen CML , de Groot DJA *et al* (2019) RAS and BRAF mutations in cell‐free DNA are predictive for outcome of cetuximab monotherapy in patients with tissue‐tested RAS wild‐type advanced colorectal cancer. Mol Oncol 13, 2361–2374. 31350822 10.1002/1878-0261.12550 PMC6822250

[11] Steendam CMJ , Atmodimedjo P , de Jonge E , Paats MS , van der Leest C , Oomen‐de Hoop E , Jansen MPHM , Del Re M , von der Thüsen JH , Dinjens WNM *et al* (2019) Plasma cell‐free DNA testing of patients with EGFR mutant non–small‐cell lung cancer: droplet digital PCR versus next‐generation sequencing compared with tissue‐based results. JCO Precis Oncol 3, 1–9. 10.1200/PO.18.00401 35100738

[12] El Messaoudi S , Rolet F , Mouliere F & Thierry AR (2013) Circulating cell free DNA: preanalytical considerations. Clin Chim Acta 424, 222–230. 23727028 10.1016/j.cca.2013.05.022

[13] Gerber T , Taschner‐Mandl S , Saloberger‐Sindhöringer L , Popitsch N , Heitzer E , Witt V , Geyeregger R , Hutter C , Schwentner R , Ambros IM *et al* (2020) Assessment of pre‐analytical sample handling conditions for comprehensive liquid biopsy analysis. J Mol Diagn 22, 1070–1086. 32497717 10.1016/j.jmoldx.2020.05.006

[14] van Dessel LF , Beije N , Helmijr JC , Vitale SR , Kraan J , Look MP , de Wit R , Sleijfer S , Jansen MP , Martens JW *et al* (2017) Application of circulating tumor DNA in prospective clinical oncology trials – standardization of preanalytical conditions. Mol Oncol 11, 295–304. 28164427 10.1002/1878-0261.12037 PMC5527445

[15] Bland JM & Altman DG (2003) Applying the right statistics: analyses of measurement studies. Ultrasound Obstet Gynecol 22, 85–93. 12858311 10.1002/uog.122

[16] R Development Core Team (2020) R: A Language and Environment for Statistical Computing. R Foundation for Statistical Computing, Vienna.

[17] Ma X , Shao Y , Tian L , Flasch DA , Mulder HL , Edmonson MN , Liu Y , Chen X , Newman S , Nakitandwe J *et al* (2019) Analysis of error profiles in deep next‐generation sequencing data. Genome Biol 20, 50. 30867008 10.1186/s13059-019-1659-6 PMC6417284

[18] Robasky K , Lewis NE & Church GM (2014) The role of replicates for error mitigation in next‐generation sequencing. Nat Rev Genet 15, 56–62. 24322726 10.1038/nrg3655 PMC4103745

[19] Jennings LJ , Arcila ME , Corless C , Kamel‐Reid S , Lubin IM , Pfeifer J , Temple‐Smolkin RL , Voelkerding KV & Nikiforova MN (2017) Guidelines for validation of next‐generation sequencing‐based oncology panels: a joint consensus recommendation of the Association for Molecular Pathology and College of American Pathologists. J Mol Diagn 19, 341–365. 28341590 10.1016/j.jmoldx.2017.01.011 PMC6941185

[20] Petrackova A , Vasinek M , Sedlarikova L , Dyskova T , Schneiderova P , Novosad T , Papajik T & Kriegova E (2019) Standardization of sequencing coverage depth in NGS: recommendation for detection of clonal and subclonal mutations in cancer diagnostics. Front Oncol 9, 851. 31552176 10.3389/fonc.2019.00851 PMC6738196

