# Corrigendum to: Comparison of variant allele frequency and number of mutant molecules as units of measurement for circulating tumor DNA

**DOI:** 10.1002/1878-0261.13086

**Published:** 2021-10-01

In [1], Christi M.J. Steendam was inadvertently omitted from the author list. CMJ Steendam was responsible for the collection of a subset of clinical samples. The corrected author list is as follows:

Manouk K. Bos, Kazem Nasserinejad, Maurice P.H.M. Jansen, Christi M.J. Steendam, Lindsay Angus, Peggy N. Atmodimedjo, Evert de Jonge, Winand N.M. Dinjens, Ron H.N. van Schaik, Marzia Del Re, Hendrikus J. Dubbink, Stefan Sleijfer, John W.M. Martens

The corrected Author contributions statement is as follows:

**Author contributions**

The corrected Author contributions statement is as follows: MB wrote the manuscript, which was corrected and approved by KN, MJ, LA, PA, EJ, WD, RS, MDR, CS, HD, SS and JM. KN performed all statistical analyses.

The corrected Conflict of interest statement is as follows:

**Conflict of interest**

The corrected Conflict of interest statement is as follows: Manouk K Bos – Research funding: Dutch Cancer Society (no. NKB‐EMCR‐2016‐108154). Maurice P. H. Jansen – Research funding: Dutch Cancer Society (no. NKB‐NKI‐2014‐7080). Christi MJ Steendam ‐ Research funding: AstraZeneca; Travel, Accommodations, Expenses: Roche, Boehringer Ingelheim, Eli Lilly. Lindsay Angus – Consulting honorarium: Merck; Speaking honorarium: Pfizer. Winand N.M. Dinjens – Consulting honorarium: Bristol‐Myers Squibb, Roche, Bayer, Astra Zeneca, Novartis. Marzia del Re – Speaker honoraria: Astellas, Astra Zeneca, Celgene, Novartis, Pfizer, Bio‐Rad, Janssen, Sanofi‐Aventis; Consulting honoraria: Ipsen, Janssen‐Cilag, Sanofi‐Aventis; Speaker's bureau: Celgene, Janssen, Sanofi; Travel support: Astra‐Zeneca, Celgene, Janssen, Bio‐Rad. Hendrikus Jan Dubbink– Supported by a grant from AstraZeneca; Consulting or Advisory Role: AstraZeneca. John W.M. Martens – Consulting honorarium: Novartis; Research funding: Cancer Genomics Netherlands (CGC.nl), funded by the Netherlands Organization for Scientific Research (NWO). Kazem Nasserinejad, Peggy Atmodimedjo, Evert de Jonge, Ron HN van Schaik, Stefan Sleijfer – No conflict of interest.

In addition, the original manuscript incorrectly stated that all lung cancer samples were collected within the START‐TKI study on lung cancer (NL58664.078.16). A subset of samples from patients with lung cancer were collected as part of routine diagnostics. As a result, the Methods section was modified, and an additional statement on the EU General Data Protection Regulation has now been added to the subsection on sample collection as follows:

**2.1 Sample collection**

Samples were obtained from the following studies: IMPACT‐CRC study on colon cancer (ClinicalTrials.gov, number NCT02117466) [10], REGORA study on colon cancer (ClinicalTrials.gov, number NCT02800330), START‐TKI study on lung cancer (CCMO number, NL58664.078.16) [11], TAX‐ESR1 study on breast cancer (trialregister.nl, number NL7280), and the CareMore‐Trastuzumab study on breast cancer (trialregister.nl, number NL4977). Additional samples from patients with lung cancer were collected as part of routine diagnostics. The study was performed in accordance with the Declaration of Helsinki and approved by the medical ethics committee of the Erasmus MC. All patients gave written informed consent prior to study procedures. In case of samples from routine diagnostics, the EU General Data Protection Regulation was applied.

Finally, the Discussion section has been adapted accordingly. The corrected last paragraph of the Discussion section is as follows:

Although our analyses were limited by the inability to analyze the accuracy of all pre‐analytical and analytical steps and their impact on agreement separately, it does provide insights into the agreement of both units of measurement in the current real‐life setting (Table 3). Although our primary aim was to investigate agreement between VAF and mutant molecules for different sequencing platforms, we lacked power to investigate differences between pre‐analytical and analytical factors among outliers. We therefore only used descriptive statistics to describe differences among pre‐analytical and analytical variables between outliers and TSVs that showed agreement. For molecular coverage however, we were able to identify a threshold that showed a high NPV. Additionally, we did not include DNA from leukocytes to exclude germline variants or variants resulting from clonal hematopoiesis [2]. Although clonal hematopoiesis is known to occur in specific genes and we reported predominantly activating, cancer‐specific hotspot mutations, a non‐malignant origin of reported TSVs cannot be excluded. All samples from cohorts that were included in this study were collected in comparable ways, and pre‐analytical and analytical methods were homogeneous within study cohorts. The methods used in all cohorts are in agreement with current guidelines and recent literature. To this end, results from our analyses reflect the accuracy of the real‐life analyses pipeline as a whole.

The above errors have no impact on the reported results and conclusions.

[The authorship has been corrected online. The original version of this paper has been updated to reflect these changes.]
