# Supplementary material for: Comparison of variant allele frequency and number of mutant molecules as units of measurement for circulating tumor DNA
Source: Mol Oncol. 2020 Oct 31;15(1):57–66. doi: 10.1002/1878-0261.12827 (PMC7782075; doi:10.1002/1878-0261.12827)
Supplement: Supplementary file 1 — Table S1. (A) Correlation among (pre)analytical factors in NGS. (B) Correlation among (pre)analytical factors in ddPCR Table S2. Pre‐analytical variables in samples containing outliers [file MOL2-15-57-s001.docx]

**Supplementary tables**

| **Table S1A. Correlation among (pre)analytical factors in NGS** | | | | | |
| --- | --- | --- | --- | --- | --- |
|  |  | **cfDNA conc** | | **MolCov** | |
|  |  | **Pearson's r** | **p value** | **Pearson's r** | **p value** |
| **Input (ng)** | Cohort 1 | 0.215 | 0.038 | 0.270 | 0.009 |
|  | Cohort 2 | 0.600 | <0.001 | 0.606 | <0.001 |
|  | Cohort 3 | NA | NA | NA | NA |
| **cfDNA conc** | Cohort 1 |  |  | 0.146 | 0.137 |
|  | Cohort 2 |  |  | 0.354 | <0.001 |
|  | Cohort 3 |  |  | 0.230 | 0.374 |
| **NA: not applicable because the factor input (ng) was constant** | | | | |  |

| **Table S1B. Correlation among (pre)analytical factors in ddPCR** | | | | | |
| --- | --- | --- | --- | --- | --- |
|  |  | **cfDNA conc** | | **MolCov** | |
|  |  | **Pearson's r** | **p value** | **Pearson's r** | **p value** |
| **Input (ng)** | Cohort 1 | 0.306 | 0.008 | 0.926 | <0.001 |
|  | Cohort 2 | 1 | <0.001 | 0.899 | <0.001 |
| **cfDNA conc** | Cohort 1 |  |  | 0.273 | 0.019 |
|  | Cohort 2 |  |  | 0.708 | <0.001 |

| **Table S2. Pre-analytical variables in samples containing outliers** | | | | | | | | |
| --- | --- | --- | --- | --- | --- | --- | --- | --- |
| **Cohort** | **SampleID** | **Outlier** | **Gene** | **Allele** | **cfDNA concentration (ng/mL)** | **Molecular coverage** | **VAF (%)** | **Mutant molecules/mL plasma** |
| Cohort1_NGS | L2011 | No | NRAS | p.Q61L | 217.8 | 6054 | 0.05 | 27 |
| Cohort1_NGS | L2011 | Upper | NRAS | p.G12C | 217.8 | 8354 | 0.04 | 27 |
| Cohort1_NGS | L2011 | Upper | EGFR | p.S464L | 217.8 | 9641 | 0.04 | 36 |
| Cohort1_NGS | L2011 | Upper | MAP2K1 | p.K57T | 217.8 | 8670 | 0.06 | 45 |
| Cohort1_NGS | L2011 | Upper | KRAS | p.Q61L | 217.8 | 8792 | 0.06 | 45 |
| Cohort1_NGS | L2011 | Upper | KRAS | p.G12S | 217.8 | 8288 | 0.07 | 54 |
| Cohort1_NGS | L2011 | No | KRAS | p.G12A | 217.8 | 8288 | 0.11 | 81 |
| Cohort1_NGS | L2011 | No | KRAS | p.Q61H | 217.8 | 8793 | 0.15 | 117 |
| Cohort1_NGS | L2011 | Upper | EGFR | p.G465R | 217.8 | 9638 | 0.16 | 135 |
| Cohort1_NGS | L2011 | No | NRAS | p.G12V | 217.8 | 8351 | 0.19 | 144 |
| Cohort1_NGS | L2011 | No | NRAS | p.G12R | 217.8 | 8354 | 0.3 | 225 |
| Cohort1_NGS | L2011 | No | NRAS | p.G12D | 217.8 | 8351 | 0.4 | 297 |
| Cohort1_NGS | L2011 | No | KRAS | p.A146T | 217.8 | 8295 | 0.61 | 459 |
| Cohort1_NGS | L2037 | No | KRAS | p.G12C | 22.5 | 3445 | 0.09 | 3 |
| Cohort1_NGS | L2037 | No | NRAS | p.Q61K | 22.5 | 1897 | 0.21 | 4 |
| Cohort1_NGS | L2037 | Lower | TP53 | p.G266R | 22.5 | 71 | 22.54 | 18 |
| Cohort1_NGS | L2052 | Lower | BRAF | p.V600E | 31.4 | 394 | 2.79 | 16 |
| Cohort1_NGS | L2065 | No | MAP2K1 | p.E203K | 13.5 | 4082 | 0.07 | 2 |
| Cohort1_NGS | L2065 | Lower | TP53 | p.G245S | 13.5 | 889 | 0.45 | 2 |
| Cohort1_NGS | L2065 | No | TP53 | p.R175H | 13.5 | 4534 | 2.58 | 70 |
| Cohort1_NGS | L2519 | Upper | PIK3CA | p.E542K | 568.2 | 4834 | 0.08 | 114 |
| Cohort1_NGS | L2519 | No | NRAS | p.Q61K | 568.2 | 1775 | 0.28 | 142 |
| Cohort1_NGS | L2519 | No | MAP2K1 | p.K57N | 568.2 | 2614 | 0.19 | 142 |
| Cohort1_NGS | L2519 | Upper | KRAS | p.G12R | 568.2 | 3029 | 0.26 | 227 |
| Cohort1_NGS | L2519 | No | EGFR | p.G465E | 568.2 | 2918 | 0.55 | 455 |
| Cohort1_NGS | L2519 | No | EGFR | p.G465R | 568.2 | 2916 | 0.62 | 511 |
| Cohort1_NGS | L2519 | No | KRAS | p.Q61H | 568.2 | 3350 | 2.39 | 2273 |
| Cohort1_NGS | L2519 | No | EGFR | p.S464L | 568.2 | 2925 | 3.69 | 3068 |
| Cohort1_NGS | L2519 | No | MAP2K1 | p.K57T | 568.2 | 2614 | 16.68 | 12386 |
| Cohort1_NGS | L2519 | No | APC | p.R1450Ter | 568.2 | 1484 | 81.94 | 34543 |
| Cohort1_NGS | L2519 | No | TP53 | p.R282W | 568.2 | 2314 | 90.41 | 59428 |
| Cohort1_NGS | L2528 | Lower | APC | p.G1312Ter | 26.5 | 137 | 12.41 | 22 |
| Cohort1_NGS | L2528 | Lower | TP53 | p.V272M | 26.5 | 62 | 30.65 | 25 |
| Cohort1_NGS | L2530 | Lower | APC | p.R876Ter | 16.3 | 574 | 0.52 | 2 |
| Cohort1_NGS | L2530 | Lower | APC | p.R1450Ter | 16.3 | 568 | 2.46 | 11 |
| Cohort1_NGS | L2530 | Lower | KRAS | p.G13D | 16.3 | 453 | 5.3 | 18 |
| Cohort2_NGS | 4 | Lower | EGFR | p.T790M | 3.3 | 189 | 2.12 | 3 |
| Cohort2_NGS | 4 | Lower | EGFR | p.L858R | 3.3 | 215 | 1.4 | 2 |
| Cohort2_NGS | 5 | Lower | TP53 | p.R273C | 2.5 | 190 | 3.68 | 4 |
| Cohort2_NGS | 5 | Lower | EGFR | p.E746_A750del | 2.5 | 231 | 8.66 | 13 |
| Cohort2_NGS | 18 | No | EGFR | p.L747_T751delinsP | 14.2 | NA | 0.24 | 4 |
| Cohort2_NGS | 18 | Lower | EGFR | p.T790M | 3.9 | 237 | 2.95 | 4 |
| Cohort2_NGS | 19 | Lower | EGFR | p.L858R | 3.9 | 238 | 4.2 | 6 |
| Cohort2_NGS | 30 | Upper | EGFR | p.E746_A750del | 199.2 | 16964 | 33.36 | 15719 |
| Cohort2_NGS | 40 | Upper | TP53 | p.R273H | 197.5 | 6410 | 0.06 | 17 |
| Cohort2_NGS | 40 | Upper | KRAS | p.G13C | 197.5 | 8964 | 19.69 | 7354 |
| Cohort2_NGS | 40 | Upper | KRAS | p.G13C | 197.5 | 11890 | 19.72 | 9771 |
| Cohort2_NGS | 49 | No | EGFR | p.T790M | 21.3 | 4184 | 0.14 | 4 |
| Cohort2_NGS | 49 | No | EGFR | p.C797S | 21.3 | 4185 | 0.14 | 4 |
| Cohort2_NGS | 49 | No | MET | p.Y1248C | 21.3 | 19093 | 8.4 | 1028 |
| Cohort2_NGS | 49 | Upper | MET | p.Y1248H | 21.3 | 19096 | 0.1 | 12 |
| Cohort2_NGS | 49 | No | MET | p.Y1248C | 21.3 | 22905 | 7.21 | 1058 |
| Cohort2_NGS | 49 | Upper | MET | p.Y1248H | 21.3 | 22919 | 0.07 | 10 |
| Cohort2_NGS | 59 | Upper | EGFR | p.T790M | 191.7 | 6651 | 0.06 | 17 |
| Cohort2_NGS | 59 | Upper | TP53 | p.R337L | 191.7 | 11610 | 0.16 | 79 |
| Cohort2_NGS | 59 | Upper | EGFR | p.E746_A750del | 191.7 | 11636 | 0.74 | 358 |
| Cohort2_NGS | 83 | No | EGFR | p.V769_D770insASV | 34.4 | 934 | 6.53 | 42 |
| Cohort2_NGS | 83 | Lower | EGFR | p.V769_D770insASV* | 34.4 | 257 | 7.39 | 13 |
| Cohort2_NGS | 83 | No | TP53 | p.M327I | 34.4 | 411 | 0.97 | 3 |
| Cohort2_NGS | 98 | Upper | KRAS | p.G13C | 352.5 | 5325 | 0.17 | 38 |
| Cohort2_NGS | 98 | Upper | KRAS | p.G13C | 352.5 | 8549 | 0.23 | 83 |
| Cohort2_NGS | 98 | Upper | EGFR | p.L747_E754delinsATSPE | 352.5 | 15005 | 0.12 | 75 |
| Cohort2_NGS | 98 | Upper | EGFR | p.L747_E754delinsATSPE | 352.5 | 19961 | 0.21 | 171 |
| Cohort2_NGS | 97 | Upper | KRAS | p.G13C | 280.0 | 3224 | 0.19 | 25 |
| Cohort2_NGS | 97 | Upper | EGFR | p.L747_E754delinsATSPE | 280.0 | 8910 | 0.17 | 63 |
| Cohort2_NGS | 122 | No | EGFR: | p.T790M | 13.3 | 1219 | 1.97 | 15 |
| Cohort2_NGS | 122 | No | EGFR: | p.C797S | 13.3 | 1219 | 1.97 | 15 |
| Cohort2_NGS | 122 | Lower | EGFR: | p.L858R | 13.3 | 68 | 7.35 | 3 |
| Cohort2_NGS | 130 | Upper | NRAS | p.G13V | 80.3 | 7228 | 0.17 | 20 |
| Cohort2_NGS | 131 | Upper | TP53 | p.Y220C | 83.3 | 9638 | 0.24 | 38 |
| Cohort2_NGS | 155 | No | EGFR | p.T790M | 17.3 | 3792 | 0.42 | 44 |
| Cohort2_NGS | 155 | No | EGFR | p.L858R | 17.3 | 3928 | 1.68 | 183 |
| Cohort2_NGS | 155 | Upper | TP53:p.G245D | | 17.3 | 4735 | 0.06 | 8 |
| Cohort2_NGS | 155 | Upper | EGFR | p.L718V | 17.3 | 6851 | 0.18 | 33 |
| Cohort2_NGS | 188 | No | EGFR | p.E746_A750del | 9.0 | 1054 | 12.24 | 83 |
| Cohort2_NGS | 188 | No | PIK3CA | p.E545K | 9.0 | 1087 | 0.28 | 2 |
| Cohort2_NGS | 188 | Lower | EGFR | p.E746_A750del | 9.0 | 145 | 17.24 | 16 |
| Cohort2_NGS | 195 | No | TP53 | p.G266V | 20.5 | 3559 | 15.71 | 358 |
| Cohort2_NGS | 195 | No | TP53 | p.E271* | 20.5 | 3560 | 30.11 | 687 |
| Cohort2_NGS | 195 | No | EGFR | p.T790M | 20.5 | 6315 | 6.56 | 1150 |
| Cohort2_NGS | 195 | No | EGFR | p.L858R | 20.5 | 6347 | 30.14 | 5314 |
| Cohort2_NGS | 195 | Upper | EGFR | p.L718V | 20.5 | 10271 | 3.7 | 1056 |
| Cohort2_NGS | 207 | No | EGFR | p.L858R | 14.3 | 581 | 4.3 | 16 |
| Cohort2_NGS | 207 | Lower | TP53 | p.V203E | 14.3 | 143 | 3.5 | 3 |
| Cohort2_NGS | 209 | No | EGFR | p.T790M | 77.7 | 7274 | 1.24 | 150 |
| Cohort2_NGS | 209 | No | EGFR | p.L858R | 77.7 | 8509 | 1.86 | 263 |
| Cohort2_NGS | 209 | Upper | TP53 | p.R213V | 77.7 | 9202 | 0.16 | 25 |
| Cohort3_NGS | L-6345 | Upper | ESR1 | p.D538G | 58.5 | 2842 | 17 | 2823 |
| Cohort3_NGS | L-6347 | Lower | SF3B1 | p.K700E | 8.3 | 269 | 17.47 | 39 |
| Cohort3_NGS | L-6347 | Lower | ESR1 | p.Y537S | 8.3 | 338 | 28.4 | 80 |
| Cohort1_ddPCR | 57 | Upper | TP53 | R273H | 91.8 | NA | 0.85 | 188 |
| Cohort1_ddPCR | 73 | Upper | PIK3CA | E545K | 210.0 | NA | 13.43 | 15549 |
| Cohort2_ddPCR | 8 | Upper | EGFR | T790M | 9.2 | NA | 8.4 | 6750 |
| Cohort2_ddPCR | 8 | Upper | EGFR | Ex19del | 9.2 | NA | 16.7 | 20750 |
| Cohort2_ddPCR | 25 | Upper | EGFR | T790M | 76.3 | 4011 | 2.2 | 1062 |
| Cohort2_ddPCR | 25 | Upper | EGFR | Ex19del | 76.3 | 4011 | 3.23 | 2237 |
| Cohort2_ddPCR | 38 | Upper | EGFR | L858R | 11.3 | 129 | 0.8 | 208 |
| Cohort2_ddPCR | 40 | Upper | EGFR | T790M | 191.7 | 6572 | 0.048 | 23 |
| Cohort2_ddPCR | 40 | Upper | EGFR | Ex19del | 191.7 | 6572 | 0.88 | 525 |
| Cohort2_ddPCR | 61 | Upper | EGFR | T790M | 118.3 | 2470 | 0.29 | 58 |
| Cohort2_ddPCR | 61 | Upper | EGFR | L858R | 118.3 | 2470 | 1.05 | 342 |
| Cohort2_ddPCR | 69 | Upper | EGFR | T790M | 190.0 | 5606 | 0.043 | 19 |
| Cohort2_ddPCR | 69 | Upper | EGFR | L858R | 190.0 | 8648 | 2.98 | 1933 |
| **NA: Not available** | | | | | | | | |
